# Identification of a Novel Single-Stranded Circular DNA Virus in Pig Feces

**DOI:** 10.1128/genomeA.00347-14

**Published:** 2014-05-01

**Authors:** Andrew K. Cheung, Terry Fei Fan Ng, Kelly M. Lager, David P. Alt, Eric L. Delwart, Roman M. Pogranichniy

**Affiliations:** a Virus and Prion Diseases Research Unit, National Animal Disease Center, USDA, Agricultural Research Service, Ames, Iowa, USA; c Infectious Bacterial Diseases Research Unit, National Animal Disease Center, USDA, Agricultural Research Service, Ames, Iowa, USA; b Blood Systems Research Institute and Department of Laboratory Medicine, University of California at San Francisco, San Francisco, California, USA; d Indiana Animal Disease Diagnostic Laboratory and Department of Comparative Pathobiology, Purdue University, West Lafayette, Indiana, USA

## Abstract

Porcine stool-associated circular virus 5 (PoSCV5) was detected in the feces of a pig with diarrhea. The complete 3,062-nucleotide genome contains two bidirectionally transcribed open reading frames (ORFs). Phylogenetic analysis of the deduced replication initiator protein (Rep) places PoSCV5 alone on a deep branch among the small circular Rep-encoding single-stranded DNA viruses.

## GENOME ANNOUNCEMENT

Metagenomic analysis of fecal samples collected from swine with diarrhea has revealed unique single-stranded (ss) circular DNA viruses (1). This report describes a new circular DNA genome from pig feces that is highly divergent from the genomes of members of the *Circoviridae*, *Geminiviridae*, or other known viral families with small circular replication initiator protein (Rep)-encoding ssDNA (CRESS-DNA) genomes (2–4). The open reading frame (ORF) arrangement can vary substantially between CRESS-DNA viruses (4). The porcine stool-associated circular virus 5 (PoSCV5) (isolate CP3) circular genome is 3,062 nucleotides (nt) in length. It contains two putative major ORFs that are bidirectionally transcribed. ORF1 is 1,617 nt in length and is predicted to encode a Rep protein involved in rolling-circle DNA replication. A BLASTp search of the deduced 355-amino acid Rep sequence indicated that the closest (yet still highly divergent) sequences belonged to the Rep-like sequences in *Entamoeba invadens* (GenBank accession no. XM 004185684, 36% identity), an uncultured marine virus (GenBank accession no. JX904139, 31% identity), and several unclassified viruses from rodent feces (GenBank accession no. JF755407, JF755416, and JF755417, 28% identity) (5). Phylogenetic analysis of the PoSV5 Rep amino acid sequence also indicated that PoSV5 does not cluster significantly with Rep from other CRESS-DNA viruses and presents itself as an isolated clade with a deep root. Motif analysis was performed by aligning its Rep sequence with those of other CRESS-DNA genomes. Putative rolling-circle replication motifs I, II, and III (LTYND, HYHLLIMC, and YVKKDGNI, respectively) and the helicase Walker A and B motifs (GETGEGKT and VIIDDFRQ, respectively) were identified. ORF2 is 1,065 nt in length. A BLAST search found no similarities for the deduced ORF2 protein. Nevertheless, we postulate that ORF2 encodes the capsid protein (Cap).

In PoSCV5, the putative ORFs are separated by a small intergenic region (SIR) of 173 nt and a larger intergenic region (LIR) of 201 nt. Transcription initiates at the SIR and terminates at the LIR. The LIR contains a stem-loop with a 14-nt loop sequence, GGGTGGGCTCTGGT, which is substantially different from the conserved nonanucleotide (TAGTATTAC) of circoviruses and geminiviruses (2, 4). Therefore, PoSV5 represents a new clade of virus associated with porcine stool with a currently unknown host species tropism. Its potential role in porcine diarrhea remains to be determined.

### Nucleotide sequence accession number.

The complete genome sequence of PoSCV5 (isolate CP3) has been deposited at GenBank under the accession no. KJ433989.
